# A review of water fluoridation implementation strategy in England 2002–2005

**DOI:** 10.1186/s12903-020-01102-w

**Published:** 2020-04-15

**Authors:** V. Wordley, R. Bedi

**Affiliations:** 1 Global Child Dental Fund, London, UK; 2 King’s College London, London, UK

## Abstract

Before 2002, there had been a long-term stalemate between private water companies and District Health Authorities across England. Between 2002 and 2005 the team in the Office of the Chief Dental Officer used leadership and advocacy skills to overcome political barriers, introducing the Water Act 2003 and a Statutory Instrument in 2005 providing indemnity for water companies. This legislation was key in removing obstacles towards the expansion water fluoridation across England.

## Background

Water fluoridation has been described as one of the top ten most important public health attainments of the twentieth century [1], with the World Health Organization recommending water fluoridation wherever it is politically and technically feasible [2].

Water fluoridation has been shown to reduce dental caries by up to 35% in deciduous teeth, sparing young children from toothache [3]. It is also a means of eliminating oral health inequalities: there is an increased likelihood of experiencing dental caries in primary teeth in the lowest social group compared to the highest [4]. Despite significant improvements in oral health in England over the past three decades, many children and adults still suffer from painful yet preventable dental disease. Inequalities persist throughout the country, with nearly a quarter of five-year-olds suffering from dental caries [5]. Debates around the expansion of water fluoridation coverage are therefore increasingly relevant.

In the United Kingdom, artificial water fluoridation benefits around 6 million people, which equates to approximately 10% national coverage. The first and largest scheme was introduced in 1964 to serve the city of Birmingham. In the following two decades this was joined by further areas in the Midlands and the North of England [6] (Table 1).

**Table 1:** Water fluoridation timeline of the UK (2012). Adapted from by the British Fluoridation Society, One in a million: The facts about water fluoridation. [Online] Available at: https://www.bfsweb.org/one-in-a-million (Accessed 18/11/2019)

City/Area in the UK	Population receiving artifically fluoridated water	Date of initiation
Cumbria	120,000	1969–71
Cheshire	137,000	1968
Tyneside	643,000	1968
Northumbria	101,000	1968
County Durham	85,000	1968
Humberside	136,000	1968/69
Lincolnshire	250,000	Mid 1970s
Nottinghamshire	287,000	Mid 1970s
Derbyshire	43,000	Mid 1970s
Birmingham	1,000,000	1964
Solihull	200,000	1964–74
Coventry	300,000	1981–89
Sandwell	300,000	1986
Dudley	305,000	1986–88
Walsall	253,000	1985–87
Wolverhampton	236,000	1986
Staffordshire	497,000	1986–88
Shropshire	22,000	1985–89
Warwickshire	431,000	1964–87
Worcestershire	253,000	1970–91
Bedfordshire	198,000	1971
TOTAL	5,797,000	

At the time that Professor Bedi was Chief Dental Officer of England (between 2002 and 2005), there had not been any major new areas fluoridated since the late 1980s. Three main barriers were preventing expansion to new areas. First was the negative academic basis for water fluoridation; second, the UK Treasury not providing the newly formed privatized water companies with indemnity should large scale claims be made by the public, and finally, there lacked clarity as to how a new scheme should be decided upon and who should take this lead: either the Local Authority or the National Health Service (NHS).

This paper will outline how the team in the Office of the Chief Dental Officer tried to overcome these and other political barriers to introduce the Water Act 2003 and a Statutory Instrument providing indemnity for water companies in 2005, legislation which significantly removed hurdles towards expanding water fluoridation across England.

## Main text

### The academic basis for water fluoridation

Concerns regarding the safety of water fluoridation have been investigated by several national and international commissions [7–9]. Relevant to the mission during 2002–2005 was the systematic York Review carried out by the NHS Centre for Reviews and Dissemination in 2000. The Review concluded that fluoridating drinking supplies reduced caries prevalence. There was no evidence of causal relationships between fluoride and systemic illnesses; the only adverse effect observed was dental fluorosis [10].

Water fluoridation is a highly contentious issue with as many lay people ardently supporting it as there are contesting it. Critics at the time recommended that further research into water fluoridation was necessary. For example, the body of work reviewed in the York Review was small and outdated with few studies following individuals longitudinally [11]. Upon these recommendations, the Department of Health (DoH) committed to further investigate the strength of the evidence surrounding water fluoridation.

In 2004 the UK Medical Research Council commissioned a follow up report in response to the recommendations of the York Review [12]. The report also found that water fluoridation was beneficial in reducing dental caries and oral health inequalities, and that there was no cause for public concern regarding health issues and water fluoridation. These two key publications served as a valid academic basis for pursuing water fluoridation as a means of eliminating enduring health inequalities across social gradients.

Separate to these reviews, in 2002, a central grant from the DoH to the British Fluoridation Society (BFS) was renewed. It was recognised that the BFS was an excellent information resource to the public and in addition, a powerful lobbying group for water fluoridation with a strong network among parliamentary members. However; the DoH also wanted a new independent group to provide information on water fluoridation. In 2004, the National Fluoride Information Centre at the University of Manchester was established with a DoH grant. The purpose of this independent body was mainly to provide independent information and help support Strategic Health Authorities as they engaged with water companies with regards to water fluoridation.

### The water act 2003

Decisions about whether to fluoridate public water supplies to help promote oral health are made at a local level by public bodies with statutory responsibilities for public health. Local authorities took these decisions up until 1974, when public health became an NHS responsibility [13]. Therefore, between 1974 and 2013 NHS health authorities made decisions on water fluoridation.

The key issue was that health authorities had been interpreting a previous 1985 Water Act as themselves having the final decision on fluoridating the water in their local communities; however, some water companies insisted that it was they who had the final discretion on implementation. This resulted in a stalemate situation where no action was being taken.

The Water Act of 2003 was an important milestone as it finally removed all ambiguities from the previous Water Fluoridation Act 1985. It clarified the stalemate situation between the two parties by establishing a statutory duty on water companies, obligating them to fully comply with any requests from health authorities for water fluoridation [14]. This removed any key decision making from water companies with regards to fluoride. This requirement remains in place today.

Successful lobbying was a key element in arriving at the point of legislation in 2003. Traditionally, the role of Chief Officers in the Department of Health has been to provide Her Majesty’s Government with advice; they have historically not been involved in lobbying. However; as media interest surrounding water fluoridation mounted, the past experience of the Chief Dental Officer was cited in the press, which certainly helped the cause. It was detailed that as a paediatric dental consultant in both fluoridated areas and non-fluoridated areas, he had personally seen a significant difference between the dental accident and emergency units in the two areas. The media attention was then heightened by a joint letter, in the Times newspaper, from three previous Secretaries of State for Health in support for water fluoridation.

Following this, the Office for the Chief Dental Officer took the unusual step of lobbying various members of government and wrote a letter to all Members of Parliament (MPs) in the House of Commons supporting water fluoridation. Furthermore, breaking with tradition, the Chief Dental Officer undertook a Question and Answer session for members of the House of Lords, presenting the case for water fluoridation prior to the vote in the Upper House. The main arguments that politicians invoked against water fluoridation included the lack of individual choice for water fluoridation being undemocratic, removing freedom of choice, in addition to environmental concerns.

The motion in both Houses of Parliament was successful in favour for water fluoridation, with the Peers in the House of Lords backing the motion by 153 votes to 31 [15]. In the House of Commons, the motion was more difficult to get through because there was vehement opposition from some MPs whose constituents did not favour water fluoridation. At one stage a compromise was being considered to allow Local Authorities and not Health Authorities to be the final decision makers. The Chief Dental Officer was asked for advice and the recommendation was made to stay with Health Authorities even if it meant risking losing the debate.

The House of Commons ultimately voted to back water fluoridation by health authorities. The Water Act 2003 passed into legislation and was thereby given royal assent. There is no doubt this is a contentious issue and careful communication and negotiation was key in gaining this result.

### Indemnity for water companies 2005

In England and Wales, the provision of water and wastewater services was moved from the public to the private sector in 1989 [16]. This established 10 private water companies who refused to extend water fluoridation unless the government gave then indemnity against future claims on any adverse impact of fluoride water. Consequently, in the years following 1989, there was a long-term stalemate between the Treasury and the DoH since no such assurance on indemnity had been made.

Water companies were concerned they would not be indemnified if the target concentration of fluoride in the water supplies exceeded the legally prescribed concentrations. There were also worries over potential liabilities and litigation from anti-fluoride opponents.

The Chief Dental Officer made personal representation to the Treasury on this important issue and after a prolonged discussion an agreement was reached. On 24th March 2005 the Parliamentary Under Secretary of State from the Department of Health published a Statutory Instrument which came into force on 1 April 2005 [17].

The Statutory Instrument stated that the Secretary of State hereby made following Regulations: that the Secretary of State agrees to indemnify a water undertaker under section 90(1) of the Water Industry Act 1991 (indemnities in respect of fluoridation), the form of that indemnity and its terms shall be those set out in Schedule 1 to these Regulations. The final barrier to extending water fluoridation was removed.

## Discussion

### Learned lessons: dental leadership and advocacy

Implementing new water fluoridation schemes will continue to be challenging, especially in this climate of powerful social media input and the growing menace of “fake news”. Anti-fluoride lobbyists now have a disproportionate voice compared to their size. Dental health professionals need to be vigilant not only in introducing new schemes but also in protecting existing ones. In addition, water fluoridation is not seen as a high priority to politicians and taking firm pro or against stances will not affect their electability.

At the Office of the Chief Dental Officer it was realised early on that the only way to achieve goals and eliminate the stalemate was via an upstream approach in central government. It was primarily through leadership and advocacy that changes materialised. Effective leadership is vital in overcoming global oral health issues; however, formal training in dental leadership at every level has not had the attention it deserves. Health care leaders must be capable of facilitating and negotiating within a competitive political environment particularly when it comes to the U. K government and an emotive topic such as water fluoridation.

Advocacy, organisation and communication skills are key characteristics underpinning leadership skills. The need for dental leadership was recognized at around the time of the water fluoridation legislation. When Professor Bedi left the Office of the Chief Dental Officer, the UK government provided a 5-year grant to establish, under his leadership, a Global Child Dental Health Taskforce. The key component of this project was to establish a Senior Dental Leadership (SDL) programme.

The objective of the SDL programme was to equip dentists in senior positions worldwide with the leadership skills necessary to effectively advocate for oral health improvement at the highest level. This includes lobbying for water fluoridation. SDL is held annually, hosted jointly between King’s College London Dental Institute and the Harvard School of Dental Medicine, with sponsorship from Colgate-Palmolive and Henry Schein. The SDL programme has been attended by over 200 of the most senior dental policy makers and dental health academic faculties from 46 countries. It celebrated its 14th successful year in 2020 [18].

## Conclusion

It was ultimately strong leadership and advocacy within dentistry and throughout the Department of Health that broke the stalemate between private water companies across England and District Health Authorities. To this day, dental caries remains a major public health threat particularly in socially deprived areas. Water fluoridation too remains a valid health intervention.

## Data Availability

Not applicable.

## Abbreviations

DoH: Department of Health
BFS: British Fluoridation Society
NHS: National Health Service
MPs: Members of Parliament
SDL: Senior Dental Leaders
